# Appreciation of different styles of humor: An fMRI study

**DOI:** 10.1038/s41598-018-33715-1

**Published:** 2018-10-23

**Authors:** Yu-Chen Chan, Wei-Chin Hsu, Yi-Jun Liao, Hsueh-Chih Chen, Cheng-Hao Tu, Ching-Lin Wu

**Affiliations:** 1 Department of Educational Psychology and Counseling, National Tsing Hua University, Hsinchu, Taiwan; 2 Research Center for Education and Mind Sciences, NTHU, Taiwan; 3 Graduate Institute of Applied Science and Technology, National Taiwan University of Science and Technology, Taipei, Taiwan; 4 Institute of Learning Sciences and Technologies, National Tsing Hua University, Hsinchu, Taiwan; 5 Department of Educational Psychology and Counseling, National Taiwan Normal University, Taipei, Taiwan; 6 Graduate Institute of Acupuncture Science, China Medial University, Taichung, Taiwan; 7 Program of Learning Sciences, National Taiwan Normal University, Taipei, Taiwan; 8 Institute for Research Excellence in Learning Sciences, NTNU, Taipei Taiwan; 9 Chinese Language and Technology Center, NTNU, Taipei Taiwan

## Abstract

Humor styles are important in facilitating social relationships. Following humor styles theory, this functional magnetic resonance imaging (fMRI) study is the first to use “one-liner” humor to investigate the neural correlates involved in appreciating humor styles that differ in terms of target (self or other) and motivation (benign or detrimental). Interestingly, we observed greater activation in response to humor that facilitates relationships with others (self-defeating and affiliative humor) than to humor that enhances the self (self-enhancing and aggressive humor). Self-defeating humor may play an important role in Chinese culture in facilitating social relationships at one’s own expense. Psychophysiological interaction (PPI) analysis revealed temporal pole (TP)-frontal functional connectivity underlying the appreciation of self-directed humor, and temporo-parietal junction (TPJ)-frontal connectivity underlying the appreciation of other-directed humor. Amygdala-frontal coupling was observed during the appreciation of detrimental humor, while nucleus accumbens (NAc)-temporal coupling and midbrain-frontal coupling appear to play a role in the affective experience of amusement in response to benign humor. This study contributes to our understanding of the neural correlates of appreciating different humor styles, including humor that facilitates social relationships.

## Introduction

In recent years, humor motivation has become a focus of substantial research interest, with particular emphasis on its role in social interaction^1,2^. Humor is a fundamental feature of social life and can be used with distinct motivations and targets. Humor is often used in a positive interpersonal manner and in contexts perceived to be playful, safe, nonserious, or, in other words, benign^3^. However, some humor expresses the motivation to disparage its target^4^, exert superiority over others^5^, or release repressed aggressive tension^6^.

The humor styles questionnaire (HSQ) developed by Martin *et al*.^7^ is a self-report measure of personality traits. It can be used to identify individual “sense of humor” differences in the use of four distinct styles of humor. These four styles are based on positions along two underlying dimensions. The first dimension is related to the intent motivating the humor and emphasizes differences in benign and detrimental humor. The second dimension is related to the target of the humor, with some humor being directed towards the self and some humor directed towards others. Based on these two dimensions, then, one’s sense of humor can be categorized in terms of four humor styles: self-enhancing humor (SE, benign humor that directly enhances the self), affiliative humor (AF, benign humor that facilitates relationships with others), self-defeating humor (SD, detrimental humor that facilitates relationships with others at one’s own expense), and aggressive humor (AG, detrimental humor that indirectly enhances the self at the expense of others)^7–11^ (see Supplementary Table S1).

In terms of navigating or negotiating social relationships, self-defeating and affiliative humor may serve to strengthen social ties and group cohesion, while self-enhancing and aggressive humor may have the opposite effect, threatening such ties. Self-defeating humor (SD) refers to humor that bolsters relationships and increases group harmony at one’s own expense. For example, an individual may use self-defeating humor to make fun of his or her own intelligence to amuse others. Affiliative humor (AF) refers to humor that bolsters relationships and increases group cohesion by making positive attributions about others. Individuals who adopt the self-defeating humor style use humor to amuse others – and to indirectly suggest a higher status for others, by implicit comparison - but this approval comes at their own expense. Individuals who engage in the affiliative humor style use humor to decrease group tension but this group cohesion comes without being harmful to oneself. Chinese society tends to be more traditional, collectivistic, face-saving, and conservative to maintain interpersonal harmony^12–15^. Since Confucian puritanism and conservatism are deeply rooted in Chinese culture, self-defeating humor may be a preferred humor style for facilitating relationships. For enhancing the self, self-enhancing humor (SE) refers to humor that directly enhances the self through humorous self-flattery, while aggressive humor (AG) refers to humor that indirectly enhances the self by suggesting a lower status for others. The nature of these styles can be seen in the following four examples, which are English translations of stimuli used in the present study:

Self-defeating (SD): “If each of my admirers were a strand of hair, I would be bald”.

Affiliative (AF): “If each of your admirers were a strand of hair, you would need two heads”

Self-enhancing (SE): “If each of my admirers were a strand of hair, I would need two heads”.

Aggressive (AG): “If each of your admirers were a strand of hair, you would be bald”.

The present study investigates the neural correlates of how people respond to different styles of humor, using participant responses to ‘one-liner’ humorous stimuli. Based on the humor styles model, the Humor Styles Questionnaire (HSQ) was developed by Martin *et al*.^7^. In the present study, however, we were interested in actual responses to humor corresponding to the different styles. Therefore, we used humorous ‘one-liners’ that were created to represent the four humor styles. This technique also has the advantage of eliciting responses without participants self-consciously reflecting upon their own responses to humor, as they do with the self-report HSQ.

Most fMRI studies have used verbal jokes to examine humor comprehension and appreciation^1,2,16,17^. Verbal jokes are composed of two components, the setup and punchline. The present study, however, uses ‘one-liners’, using the technique of exaggeration to make the one-liners funny. We make pairwise comparisons using four carefully matched humor styles and a non-humor baseline with our humorous one-liner stimuli. Based on the definitions of the four styles of humor identified by Martin *et al*.^7^, this fMRI study is the first to use one-liner verbal humor to identify the neural correlates of humor appreciation in responses to humor styles differing in terms of their underlying motivation and their overt target.

Several fMRI studies using verbal jokes or riddles have examined brain regions to investigate humor processing (e.g., incongruity, resolution and appreciation)^16–18^, humor structure (e.g., logical mechanisms or humor techniques)^19–21^, humor content (e.g., hostile jokes)^2^, humorlessness^1^, and sex/gender differences in humor^22^. Existing studies of socially-directed aggressive humor have focused primarily on humor targeted toward others^1,2^. The present study of the neural correlates underlying appreciation of different styles of humor, by contrast, also focuses on self-defeating humor (SD), which may be of particular relevance in a Chinese cultural setting. One earlier study attempted to explore humor styles using point-to-self and point-to-other humor via verbal jokes^23^. The present study seeks to extend this and similar attempts^1,2,23^ by identifying the neural correlates of humor appreciation for humor with different targets (self/others) and motivations (detrimental/benign).

Humor processing involves cognition, affect, and laughter^22,24^ and much has been learned in recent years about the neural regions involved in these processes^16,17,22^. The dopaminergic midbrain, including the ventral tegmental area (VTA) and substantia nigra (SN), receives information and regulates motivated behavior^25^. The prefrontal cortex (PFC), particularly the medial prefrontal cortex (mPFC), plays a vital role in social cognition and socio-emotional processing^1,2^. The interaction of funniness and the social appropriateness of humor involves the vmPFC^26^. The mesocorticolimbic system (MCL) and mesolimbic dopaminergic reward system have been implicated in humor appreciation and positive affect, i.e., experiencing the feeling of amusement from understanding a joke^1,16^. An earlier study of our own using exaggeration jokes found that amusement was associated with activity in the amygdala, inferior parietal lobe and inferior frontal gyrus^20^. In previous humor motivation studies, detrimental verbal jokes (i.e., hostile jokes) and benign verbal jokes (i.e., non-hostile jokes) have been shown to engage a network involving cognitive processing in the PFC and humor appreciation processing in the midbrain, ventral striatum (e.g., nucleus accumbens, NAc), limbic connections in the amygdala, and paralimbic connections in the anterior cingulate cortex (ACC) and orbitofrontal cortex (OFC)/ventromedial prefrontal cortex (vmPFC)^1,2^.

In addition, previous studies of theory of mind (ToM) involving humor appreciation have suggested that the feeling of amusement is associated with the MCL system in the temporo-parietal junction (TPJ), the middle temporal gyrus (MTG), and the OFC^20^. Humor intensity has been associated with increased activation in the TPJ, temporal pole (TP) (BA 38), and MCL system^27^. Regarding the social motivation of humor, inferencing often requires attributing intentions to “others”. One of our own earlier studies using bridging-inference jokes found that ToM-related processing was associated with activity in the TPJ, MTG, and the OFC^20^.

In our previous study of humor motivation, benign verbal humor (i.e., non-hostile jokes) primarily showed increased activation in the NAc and midbrain, while detrimental verbal humor (i.e., hostile jokes) was associated with increased activation in the dorsomedial PFC (dmPFC) and midbrain^2^. The present study further investigates the neural correlates underlying the appreciation of humor that differs in terms of motivation (benign and detrimental) and target (self and others). We focus on regions of interest (ROIs) in the dopaminergic pathway (midbrain and NAc), limbic system (amygdala), paralimbic cortex (TP [anterior superior temporal gyrus], ACC, and OFC/vmPFC), and regions involved with ToM (TPJ and MTG) and cognitive processing (PFC). In addition, we employ psychophysiological interaction (PPI) analysis to examine the functional connectivity of these ROIs during the appreciation of humorous one-liners. Based on previous humor studies^1,2,16–24,27,28^, PPI analyses primarily sought to identify inter-regional interactions using the NAc, midbrain, TPJ, amygdala, and TP as the seed regions.

Based on previous humor studies on benign verbal jokes (non-hostile jokes) in humor motivation studies^1,2^ and bridging-inference jokes^20^, we predicted that the NAc and midbrain would engage distinct MCL and ToM networks to process benign, other-directed humor, as such humor involves understanding others’ intentions. For detrimental verbal jokes (e.g., hostile jokes)^1,2^ and exaggeration jokes^20^, we predicted that the functional coupling between the amygdala and frontal circuits would predict positive affect for detrimental humor. Finally, we predicted that the TP functional coupling with frontal regions would predict intensity^27^ of positive affect for self-directed humor.

## Results

### Behavioral data

Participants rated the funniness of each condition on a 4-point scale (1 = not funny at all, 2 = not funny, 3 = funny, 4 = very funny) during the scanning procedure. Nonparametric Kruskal-Wallis one-way analysis of variance by ranks tests were performed on the funniness ratings for the four types of humor and the non-humor baseline. Ratings were found to differ significantly across the five conditions, χ^2^(4) = 112.712, *p* < 0.001. A post hoc test showed that the four humor conditions were significantly funnier than the non-humor condition.

### fMRI results

The main effects of target, the main effects of motivation (Table 1), the interaction between target and motivation (Table 1), and simple main effects (Table 2) were examined.

**Table 1:** Activated brain regions for the main effects and interaction between target (self/others) and motivation (benign/detrimental humor).

Brain region	MNI coordinates			BA	Voxels	Z score
	*x*	*y*	*z*			
**Main effects for target**						
*Self-directed humor* > *other-directed humor*						
No significant differences						
*Other-directed humor* > *self-directed humor*						
Nucleus accumbens (NAc)	−4	8	0		9	3.29
Midbrain	18	−20	−8		17	3.17
**Main effects for motivation**						
*Benign humor* > *detrimental humor*						
Temporo-parietal junction (TPJ)	50	−72	16	39	13	3.31
*Detrimental humor* > *benign humor*						
No significant differences						
**Interaction of target and motivation**						
Nucleus accumbens (NAc)	6	10	−2		69	6.61
sgACC	6	12	−8	25	53	6.52
sgACC	−2	6	−4	25	48	5.85
pgACC	−8	42	8	32	11	3.69
Midbrain	10	−18	−18		48	5.92
Temporal pole (TP)	−50	12	−24	38	23	5.37
Middle temporal gyrus (MTG)	60	−2	−22	21	38	4.74
MTG	−48	10	−28	21	11	4.73
Amygdala	−24	−4	−20		19	4.38

Note: The activation threshold was set at *p* < 0.05 and FWE (familywise error rate) corrected at the peak level, and clusters greater than or equal to 8 are presented.

**Table 2:** Activation levels of ROIs in the brain showing simple main effects during humor appreciation.

Brain region	*Facilitating relationships*									
	*Self-defeating humor (SD)*					*Affiliative humor (AF)*				
	MNI coordinates			Voxels	Z-value	MNI coordinates			Voxels	Z-value
	x	y	z			x	y	z		
**Target**	**Self-directed humor (SD** > **SE)**					**Other-directed humor (AF** > **AG)**				
Temporal pole (TP) (BA 38)	−50	12	−24	24	4.92					
Midbrain	0	−24	−12	88	4.80					
	12	−16	−18	23	4.30					
Nucleus accumbens (NAc)	6	4	0	36	4.25	6	10	−6	26	4.42
Subgenual anterior cingulate cortex (sgACC) (BA 25)						2	4	−6	86	4.92
Temporo-parietal junction (TPJ) (BA 39)						38	−56	22	31	4.85
						−48	−66	28	10	3.33
Medial orbitofrontal cortex (mOFC) (BA 11)						4	54	−10	9	3.50
**Motivation**	**Detrimental humor (SD** > **AG)**					**Benign humor (AF** > **SE)**				
NAc						10	10	0	55	5.04
						−4	6	−2	32	4.88
Middle temporal gyrus (MTG) (BA 22/21)						−54	−48	0	64	4.40
Midbrain						10	−18	−18	27	4.38
						−2	−24	−14	81	4.36
Ventromedial prefrontal cortex (vmPFC) (BA 10)						8	64	6	14	4.07
TP						−50	12	−24	13	3.67
TPJ						−44	−56	12	16	3.25
sgACC	4	16	−6	45	4.89					
Amygdala	−26	−6	−22	8	4.02					

Note: MNI coordinates of peaks of relative activation within ROI regions activated by simple main effects of self-directed humor, other-directed humor, detrimental humor, and benign humor. The activation threshold was set to *p* < 0.05 FWE (familywise error rate) corrected at the peak level, and clusters greater than or equal to 8 are presented. SD = self-defeating humor; SE = self-enhancing humor; AF = affiliative humor; AG = aggressive humor.

#### Main effects of target (self-directed humor versus other-directed humor)

The contrast of self-directed humor (SE + SD) versus other-directed humor (AF + AG) showed no significant activation.

#### Main effects of target (other-directed humor versus self-directed humor)

The contrast of other-directed humor (AF + AG) versus self-directed humor (SE + SD) showed greater activation in the left NAc and right midbrain (Table 1) during appreciation of other-directed humor.

#### Main effects of motivation (benign humor versus detrimental humor)

The contrast of benign humor (SE + AF) versus detrimental humor (SE + AG) showed greater activation in the right TPJ (Table 1) during appreciation of benign humor.

#### Main effects of motivation (detrimental humor versus benign humor)

The contrast of detrimental humor (SD + AG) versus benign humor (SE + AF) showed no significant activation.

#### Interaction between target and motivation

An interaction between motivation and target was observed in the activation of the right NAc, bilateral sgACC (BA 25), right midbrain (including SN and VTA), left temporal pole (TP) (BA 38), bilateral MTG, and left amygdala (Table 1).

#### Simple effect of self-directed humor (SD > SE)

**Figure 1:**
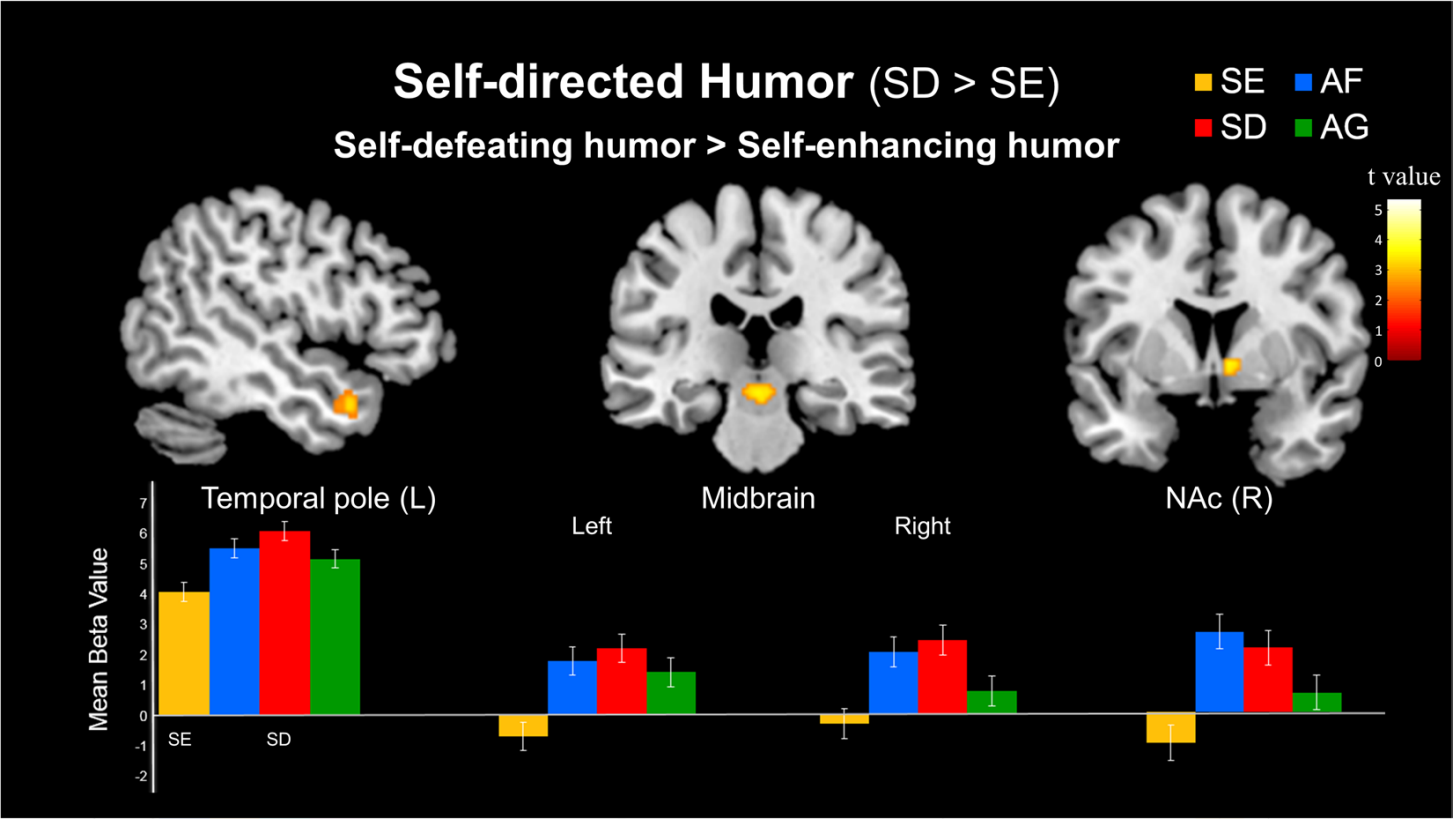
Simple effects of the differential neural mechanisms related to self-directed humor. (Top) Stronger responses to self-defeating humor (SD) than to self-enhancing humor (SE) in the left temporal pole, bilateral midbrain, and right nucleus accumbens (NAc). (Bottom) Parameter estimates (SPM betas) are plotted for these four regions. Standard error of the mean (SEM) bars are shown.

In the self-directed humor conditions, the contrast between the self-defeating humor (SD) and self-enhancing humor (SE) conditions revealed greater activation during the appreciation of self-defeating humor in the TP (BA 38), including the anterior STG (BA 22) (−50, 12, −24; Z = 4.92; 24 voxels) and anterior MTG (BA 21) (−48, 10, −28, Z = 3.90; 11 voxels). Subcortically, significantly increased activity was also observed in the bilateral midbrain, including the SN (12, −16, −18; Z = 4.30; 20 voxels) and VTA (0, −24, −12; Z = 4.80; 76 voxels). In addition, the right NAc was activated (Table 2 and Fig. 1).

#### Simple effect of self-directed humor (SE > SD)

In the self-directed humor conditions, no significant activity was found in the contrast between self-enhancing (SE) and self-defeating (SD) humor.

#### Simple effect of other-directed humor (AF > AG)

**Figure 2:**
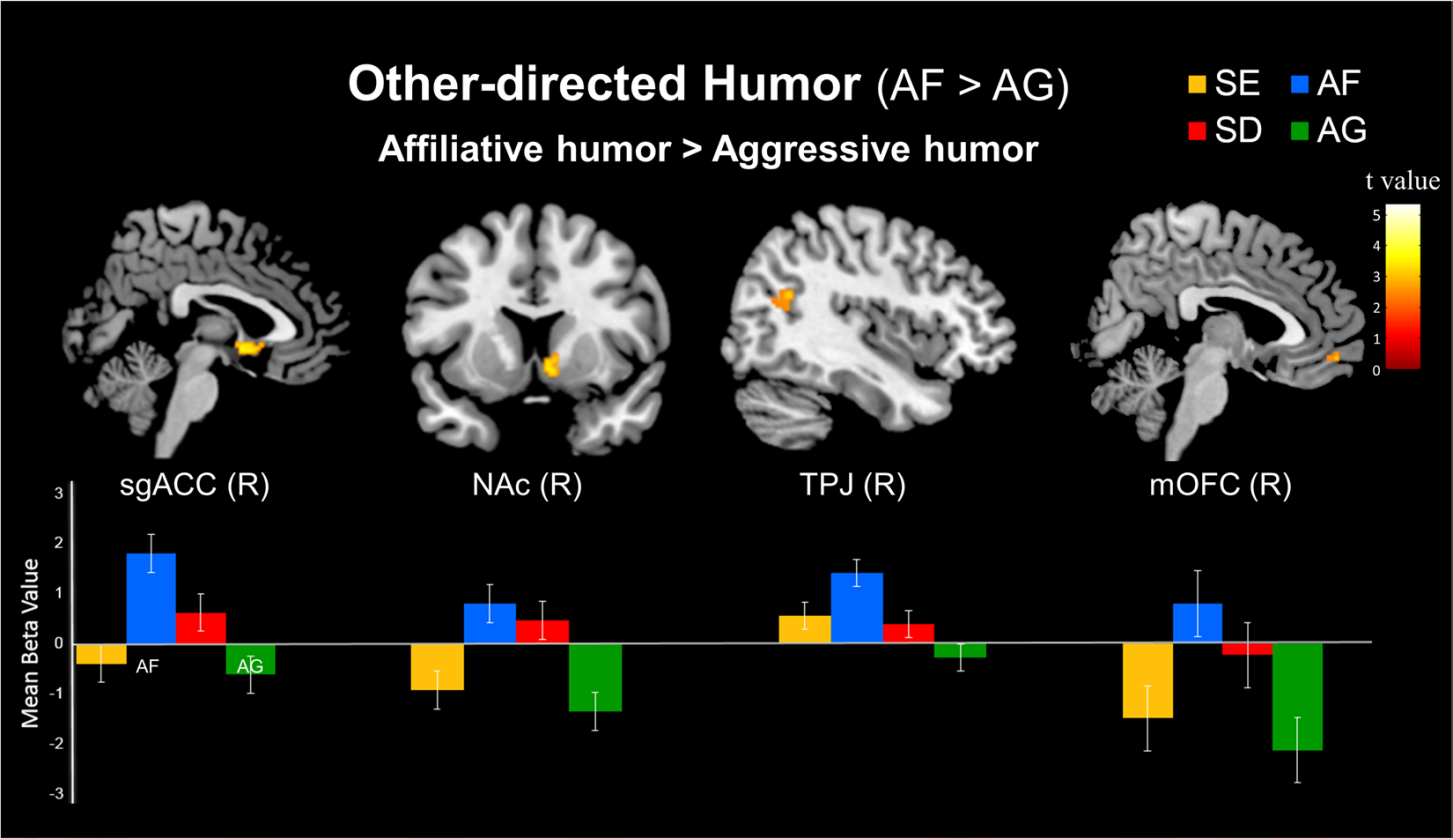
Simple effects of the differential neural mechanisms related to other-directed humor. (Top) Stronger responses to affiliative humor (AF) than to aggressive humor (AG) in the right subgenual anterior cingulate cortex (sgACC), right nucleus accumbens (NAc), right temporoparietal junction (TPJ), and right medial orbitofrontal cortex (mOFC). (Bottom) Parameter estimates (SPM betas) are plotted for these four regions. Standard error bars are shown.

In the other-directed humor conditions, the contrast between the affiliative humor (AF) and aggressive humor (AG) conditions showed greater activation during the appreciation of affiliative humor in the right sgACC, right NAc, bilateral TPJ (BA 39), and right mOFC (BA 11) (Table 2 and Fig. 2).

#### Simple effect of other-directed humor (AG > AF)

In the other-directed humor condition, the contrast between the aggressive humor (AG) and affiliative humor (AF) conditions showed greater activation during the appreciation of aggressive humor in the right midbrain (MNI = 4, −42, −24; Z = 4.22; 10 voxels).

#### Simple effect of detrimental humor (SD > AG)

**Figure 3:**
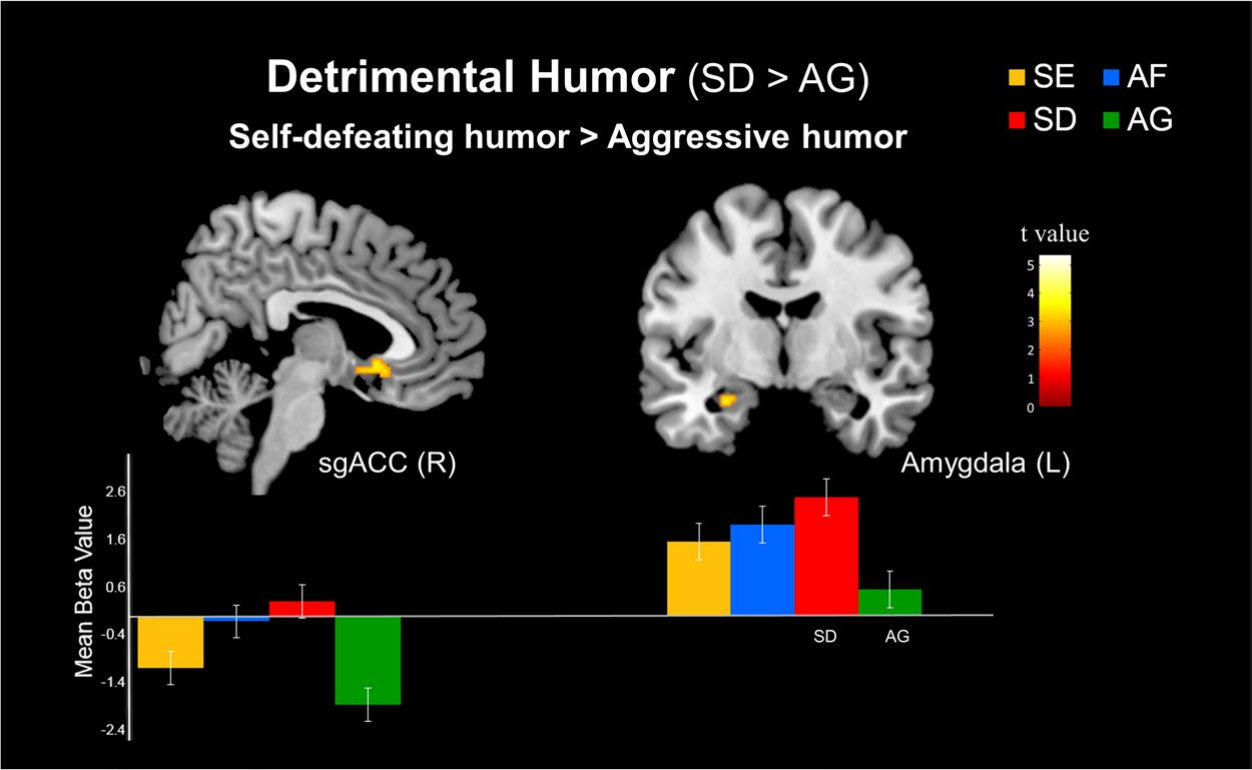
Simple effects of the differential neural mechanisms related to detrimental humor. (Top) Stronger responses to self-defeating humor (SD) than to aggressive humor (AG) in the right subgenual anterior cingulate cortex (sgACC) and left amygdala. (Bottom) Parameter estimates (SPM betas) are plotted for the two regions. Standard error bars are shown.

In the detrimental humor conditions, the contrast between the self-defeating humor (SD) and aggressive humor (AG) conditions demonstrated greater activation during the appreciation of self-defeating humor in the right sgACC (BA 25) and left amygdala (Table 2 and Fig. 3).

#### Simple effect of detrimental humor (AG > SD)

In the detrimental humor conditions, no significant activity was found in the contrast between the aggressive (AG) and self-defeating (SD) humor.

#### Simple effect of benign humor (AF > SE)

**Figure 4:**
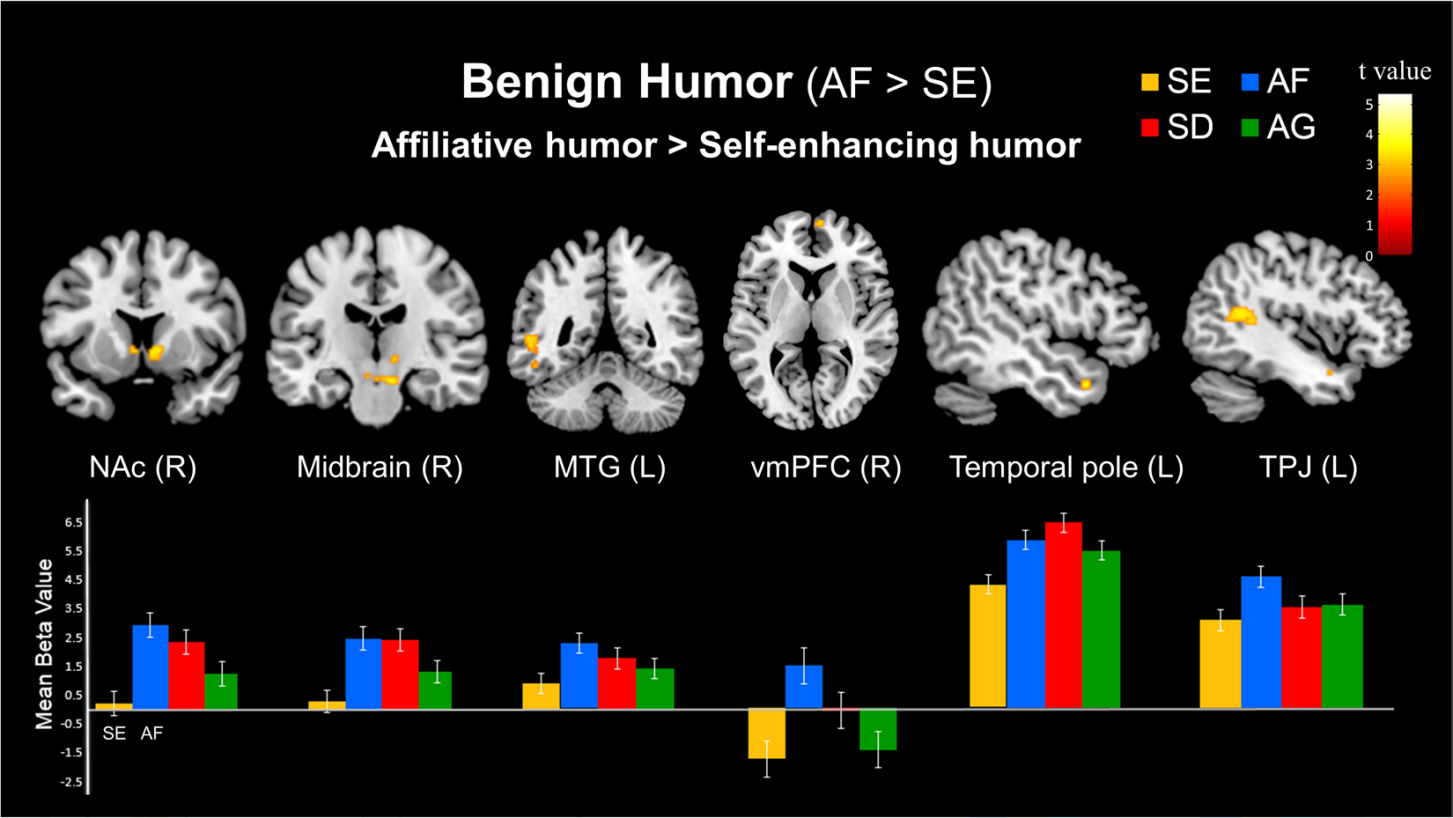
Simple effects of the differential neural mechanisms related to benign humor. (Top) Stronger responses to affiliative humor (AF) than to self-enhancing humor (SE) in the right nucleus accumbens (NAc), right midbrain, left middle temporal gyrus (MTG), right ventromedial prefrontal cortex (vmPFC), left temporal pole, and left temporoparietal junction (TPJ). (Bottom) Parameter estimates (SPM betas) are plotted for these six regions. Standard error bars are shown.

In the benign humor conditions, the contrast between the affiliative humor (AF) and self-enhancing humor (SE) conditions showed greater activation during the appreciation of affiliative humor in the bilateral NAc, left MTG (BA 22/21), bilateral midbrain, including SN (10, −18, −18; Z = 4.38, 27 voxels), VTA (10, −16, −18; Z = 4.30; 20 voxels) and VTA (−2, −24, −14; Z = 4.37; 81 voxels). In addition, the right vmPFC (BA 10), left TP (BA 38) and left TPJ (BA 39) were activated (Table 2 and Fig. 4).

#### Simple effect of benign humor (SE > AF)

In the benign humor conditions, no significant activity was found in the contrast between the self-enhancing (SE) and affiliative (AF) humor.

### Functional connectivity: psychophysiological interaction analysis (PPI)

Based on our simple main effect results, we observed greater activation in response to humor that facilitates relationships (self-defeating and affiliative humor) than to humor that enhances the self (self-enhancing and aggressive humor) (Table 2). PPI analysis was further conducted to determine whether an interaction exists between a psychological variable (motivation and target of humor) and the functional coupling of brain areas in facilitating relationships. The PPI analyses primarily sought to identify inter-regional interactions using the NAc, midbrain, TPJ, amygdala, and TP as the seed regions. (1) For the self-directed humor styles, PPI analysis using the left TP (−50, 12, −24) as a seed showed enhanced functional connectivity with the left OFC in the comparison between the self-defeating humor and the self-enhancing humor. In addition, using the right NAc (6, 4, 0) as a seed revealed functional connectivity with the right midbrain. (2) For the other-directed humor styles, PPI analysis using the left TPJ (−48, −66, 28) as a seed showed functional connectivity with the right MFG in the comparison between the affiliative humor and aggressive humor. In addition, using the NAc as a seed showed functional connectivity with the left MTG. (3) For the detrimental humor styles, PPI analysis using the amygdala (−26, −6, 22) as a seed showed functional connectivity with the frontal cortex (dlPFC and dmPFC) in the comparison between the self-defeating humor and aggressive humor. (4) For the benign humor styles, PPI analysis using the right NAc (10, 10, 0) as a seed showed functional connectivity with the right TP in the comparison between the affiliative humor and self-enhancing humor. In addition, using the left midbrain (−2, −24, −14) as a seed showed functional connectivity with the left frontal pole. Using the right midbrain (10, −18, −18) as a seed showed functional connectivity with the right MTG (uncorrected). Finally, using the left TP (−50, 12, −24) as a seed showed functional connectivity with the left MTG and left frontal pole (Table 3).

**Table 3:** Functional connectivity of the seeds of psychophysiological interaction (PPI) analyses.

Anatomical region	BA	Voxels	Side	MNI coordinates			Z score
				*x*	*y*	*z*	
(1) *Self-directed humor (SD* > *SE)*							
**TP seed (−50, 12, −24)**							
lOFC	11	91	L	−30	50	−10	4.97
**NAc seed (6, 4, 0)**							
Midbrain		11	R	6	−38	−20	3.14
(2) *Other-directed humor (AF* > *AG)*							
**NAc seed (6, 10, −6)**							
MTG	21	36	L	−58	−2	−20	3.39
**TPJ seed (**−**48**, −**66, 28)**							
Middle frontal gyrus (MFG)	6	15	R	36	−6	60	2.97
(3) *Detrimental humor (SD* > *AG)*							
**Amygdala (**−**26**, −**6**, −**22)**							
Medial frontal gyrus (dlPFC)	9	31	R	12	50	30	3.36
Medial frontal gyrus (dmPFC)	9	22	L	−8	46	32	3.17
(4) *Benign humor (AF* > *SE)*							
**NAc seed (10, 10, 0)**							
Temporal pole (BA 38)	38	11	R	46	8	−12	3.37
**Midbrain seed (**−**2**, −**24**, −**14)**							
Frontal pole (SFG)	10	30	L	−26	52	−2	3.51
**Midbrain seed (10**, −**18**, −**18)**							
MTG^†^ (uncorrected)	21	10	R	56	6	−16	2.96
**TP seed (**−**50, 12**, −**24)**							
MTG	21	25	L	−62	−26	−14	3.58
Frontal pole (MFG)	10	43	L	−40	48	14	4.51

Note: The activation threshold was set at *p* < 0.05 and FWE (familywise error rate) corrected at the peak level, and clusters greater than or equal to 8 are presented.

### Correlational analysis data

**Figure 5:**
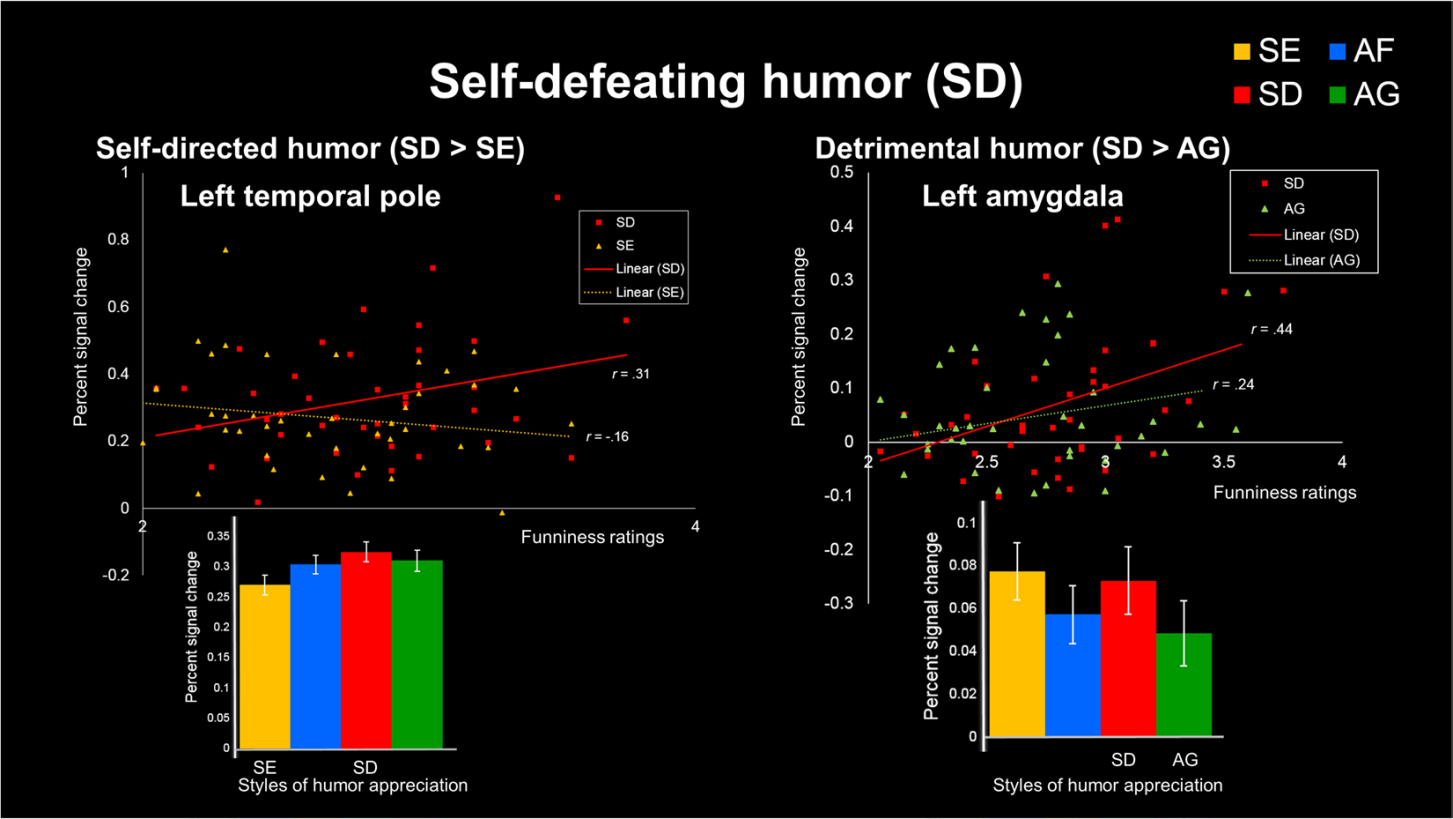
BOLD activity correlation with self-defeating humor appreciation. (Top left) Scatter plot illustrating the positive correlation between funniness ratings for self-defeating one-liners and activation of the left temporal pole (TP) (*r* = 0.31, *p* < 0.05). Negative correlation between self-enhancing humor (SE) appreciation ratings and no significant activation of the left TP (*r* = −0.16, *p* > 0.05). (Bottom left) Percent signal change in relation to humor appreciation for processing of SD and SE in the left TP. (Top right) Scatterplot illustrating the positive correlation between funniness ratings for SD one-liners and activation of the left amygdala (*r* = 0.44, *p* < 0.01). Positive correlation between aggressive humor (AG) appreciation ratings and no significant activation of the amygdala (*r* = 0.24, *p* > 0.05). (Bottom right) Percent signal change in relation to humor appreciation for processing of SD and AG in the left amygdala.

Based on our results, greater activation was observed during appreciation of detrimental humor targeted at the self (self-defeating humor) and benign humor directed at others (affiliative humor) (Tables 2 and 3). The present study further analyzed the correlations between self-defeating humor and subjective funniness scores as well as affiliative humor and subjective funniness scores. Based on TP-frontal connectivity found during the appreciation of self-directed humor, activity in the TP region was chosen. Based on amygdala-frontal connectivity identified during the appreciation of detrimental humor, activity in the amygdala region was chosen. By contrast, activity in the TPJ region was chosen for other-directed humor and activity in the NAc region was chosen for benign humor. In terms of self-directed humor (the SD versus SE contrast), activity in the left TP correlated positively with funniness ratings (*r* = 0.31, *p* = 0.048) for self-defeating humor. For detrimental humor (the SD versus AG contrast), activity in the left amygdala correlated positively with funniness (*r* = 0.44, *p* = 0.004) for self-defeating humor. No correlations were noted between funniness scores and brain regions (TP and amygdala) during the appreciation of self-enhancing humor (*r* = −0.16, *p* = 0.316), nor during the appreciation of aggressive humor (*r* = 0.24, *p* = 0.133) (Fig. 5). No correlations were noted between funniness scores and brain regions (TPJ and NAc) during the appreciation of affiliative humor.

## Discussion

Humor plays an important role in negotiating social relationships and people adopt particular styles of humor to serve this purpose. These different styles are defined by whether humor is directed at one’s self or at others, and whether it is benign or detrimental. In this study, we used one-line humor stimuli in an attempt to advance our understanding of the neural correlates underlying humor appreciation for these different styles of humor. To our knowledge, this present event-related fMRI study is the first in which stimuli were categorized based on the humor styles identified by Martin *et al*.^7^ using one-liner humor instead of the self-report measure, the HSQ.

We identified an interaction between motivation and target. Interestingly, we observed greater activation in response to humor that facilitates relationships (self-defeating and affiliative humor) than to humor that enhances the self (self-enhancing and aggressive humor) (Table 2). Humor that facilitate relationships tends to bolster social relationships and thereby increase group cohesion. Within the context of Chinese-culture, self-defeating humor (which enhances the status of others, by implicit comparison) may be of particular importance. In addition, the results of this study are consistent with previous studies, in that these brain regions exhibited more activation in response to benign humor than to detrimental humor^1,2^.

Previous studies have yielded inconsistent results regarding the mental representation of self and others^29^. The mental representations of both the self and others have been associated with activity in the TP and TPJ^29^. The TP and TPJ have been implicated in inferential processing and episodic memory recall, suggesting that these processes are involved in mentally representing one’s self and others^29^. In the present study, the appreciation of both self-directed and other-directed humor had distinct neural mechanisms in the TP and TPJ.

PPI analyses further demonstrated the inter-regional co-activation (functional coupling) of different brain regions during the appreciation of different styles of humor. The TP showed greater functional connectivity with the OFC during self-defeating humor than during self-enhancing humor, while neural signaling was observed in the TPJ-MFG coupling during the appreciation of other-directed humor (AF > AG). The finding thus seems to further distinguish the roles of the TP-PFC (OFC) for self-directed humor and TPJ-PFC (MFG) for other-directed humor.

One earlier study found benign verbal humor (i.e., non-hostile jokes) to be associated with increased activation in the amygdala, midbrain, NAc, and vmPFC, while detrimental verbal humor (i.e., hostile jokes) was associated with increased activation in the midbrain and dorsomedial PFC (dmPFC)^2^. In the present study, using one-liners, benign humor was associated with increased activation in the NAc, midbrain, vmPFC, and TPJ, whereas detrimental humor was associated with increased activation in the sgACC and amygdala. PPI analysis demonstrated amygdala coupling with the PFC (including the dlPFC and the dmPFC) for detrimental humor, while neural signaling was observed in the NAc coupling with the TP for benign humor. In addition, midbrain activity covaried with PFC activity and the TP exhibited coupling with the MTG and PFC for benign humor. These findings highlight the importance of limbic-frontal connectivity during the appreciation of detrimental humor (SD > AG) and mesolimbic-temporal-frontal connectivity during the appreciation of benign humor (AF > SE).

The mesolimbic reward centers for humor were associated with activity in the NAc, midbrain, and amygdala during the appreciation of different types of humor^1,2,16,20^. For self-defeating humor, the present study revealed a distinction between TP-frontal connectivity for appreciating self-directed humor (SD > SE) and amygdala-frontal connectivity for appreciating detrimental humor (SD > AG). Conversely, for affiliative humor, the study also revealed a distinction between TPJ-frontal connectivity for appreciating other-directed humor (AF > AG) and NAc-temporal connectivity, midbrain-frontal connectivity and TP-temporal-frontal connectivity for appreciating benign humor (AF > SE).

The present study supports the importance of the NAc and midbrain in appreciating benign humor. However, the involvement of the amygdala may be related to its role in resolving the incongruity with “getting” a joke and then “enjoying” it, specifically at the expense of the self. Self-defeating humor can make individuals happy by improving interpersonal relationships^30^. The experience of amusement from appreciating self-defeating humor may involve the amygdala. The amygdala is involved in pleasurable experiences^1,2,16,20^ and evaluative processes associated with socially and biologically relevant emotions^31,32^. In contrast, the NAc and midbrain mediate evaluative processes associated with reward, motivation, and approach behavior^33^. Amygdala activation has been implicated in the moment-to-moment experiencing of emotionally arousing events and is associated with later emotional memory for emotional experiences^34^. This study links responses to self-defeating humor in the amygdala with socially and biologically relevant emotions and past emotional memories.

Self-defeating humor attempts to amuse others at one’s own expense. Within the context of Chinese culture, self-defeating humor might facilitate cohesion and interpersonal harmony in social interactions. In this study, based on responses to one-liner humor instead of the self-report HSQ, increased activation was observed in the amygdala during the appreciation of self-directed detrimental humor (SD) but not other-directed detrimental humor (AG).

This study revealed a positive correlation between the percent signal change in the left TP during self-directed humor (self-defeating humor versus self-enhancing humor) and subjective funniness scores. Additionally, correlation analysis during detrimental humor (self-defeating humor versus aggressive humor) revealed that increased activity in the left amygdala was associated with higher funniness scores. In the appreciation of self-defeating humor, participants showed a positive correlation between activity in the TP and amygdala and subjective funniness ratings, suggesting involvement of these regions in the experience of amusement.

In summary, TP-frontal connectivity was involved in self-directed humor, probably reflecting the importance of self-representation processing for this style of verbal humor. TPJ-frontal connectivity was particularly active for other-directed humor, suggesting the importance of ToM for understanding the mind of others. Additionally, amygdala-frontal connectivity appears to subserve affective appreciation of detrimental humor, whereas the NAc-temporal connectivity and midbrain-frontal connectivity appear to play a role in the affective experience of amusement in response to benign humor.

Self-defeating humor appears to facilitate cohesion and interpersonal harmony in Chinese culture. Our finding of a positive correlation between funniness ratings and TP and amygdala activity underlying the appreciation of self-defeating humor (at one’s own expense) may be consistent with this suggestion. PPI results further confirmed functional coupling between the TP-OFC for self-directed humor and amygdala-PFC for detrimental humor underlying the appreciation of self-defeating humor. Future studies might further examine sex/gender differences in particular humor styles, specifically in appreciating self-defeating humor.

## Methods

### Participants

Participants were 42 right-handed healthy adults (21 females) aged between 20 and 30 years (23.95 ± 2.81). All participants were native Mandarin speakers with no history of neurological or psychiatric problems. Right-hand dominance was indicated by the Edinburgh Handedness Inventory^35^. All experimental protocols performed in this study were approved by the Research Ethics Committee of National Tsing Hua University (NTHU) in Taiwan. Written informed consent was obtained. All research was performed in accordance with relevant guidelines and regulations.

### Stimuli

In the present study, we developed one-line humor stimuli based on the model of humor styles by Martin *et al*.^7^. In contrast to earlier research using the self-report HSQ, the present study used humorous one-liners along with matched stimuli, non-humorous one-liners. The corresponding baseline non-humorous one-liner stimuli were constructed with neutral sentences of matching length and punctuation. We used exaggeration to develop the humorous one-liner stimuli, in which some element of a situation was exaggerated in terms of quality or quantity to such an extreme as to violate common sense understanding^20,22^. In our previous fMRI studies of verbal humor^1,2,16,17,20,22^, stimuli included verbal jokes composed of two components, the setup and punch line. However, unlike our previous studies^18^, the present study used one-liner humor with the exaggeration technique, which conceptually overlaps with nonsense or absurd humor. The stimuli are described in greater detail in the supplementary data (see Supplementary Table S2).

Seven hundred stimuli were initially chosen from our database. All stimuli were created by members of the research team. We performed two behavioral studies to choose the stimuli for the main fMRI study. In the first behavioral study, the material for each condition was 140 sentences, with 700 sentences used in total. Participants rated stimuli printed on paper, classified the stimuli and rated the degree of comprehensibility, funniness, and exaggeration on a 9-point scale. Classification refers to categorization of the humor into one of the five categories (SD, SE, AF, AG or not funny). Comprehensibility denotes how well participants understood the stimuli. Funniness indicates how amused participants were. Exaggeration refers to how unexpected the one-liner stimuli were to participants. For classification, the overall rate of correct responses was 90.43%, indicating that participants could correctly judge the five types of stimuli. The mean comprehensibility rating was 8.32 ± 0.62, indicating that participants could perceive what the stimuli were intended to express. The mean funniness rating was 5.26 ± 2.29, meaning that the participants found the one-liner humor amusing. Based on the ratings, we chose 135 sentences to represent each type of stimuli, for a total of 675 sentences (see Supplementary Table S3). In the second behavioral study, every participant rated 135 sentences, including sentences for four humor styles and one-liner nonhumor (baseline). Participants read the one-liner stimulus and pressed the space bar once they understand the stimulus. Participants then classified the stimuli and rated the levels of comprehensibility, funniness, and exaggeration on a 7-point scale (see Supplementary Table S4). A Chi-square test showed that the percentage of categories chosen was significantly different among the five types of stimuli, χ^2^(20) = 28723.702, *p* < 0.001. A post hoc test shows that participants correctly classified all five types of stimuli (see Supplementary Table S5).

Based on the ratings of the two behavioral studies, we chose 100 sentences to represent each type of stimuli, with a total of 500 sentences used during the fMRI study. The mean and standard deviation of reaction time for understanding one-liner stimuli was 4892.45 ± 2196.79 ms. The length of each sentence was 19 to 24 Chinese characters (21.77 ± 1.16). The procedure for selecting the stimuli and the results as well as post-scan ratings are described in greater detail in the supplementary data (see Supplementary Tables S3–S7).

### Experimental paradigm

**Figure 6:**
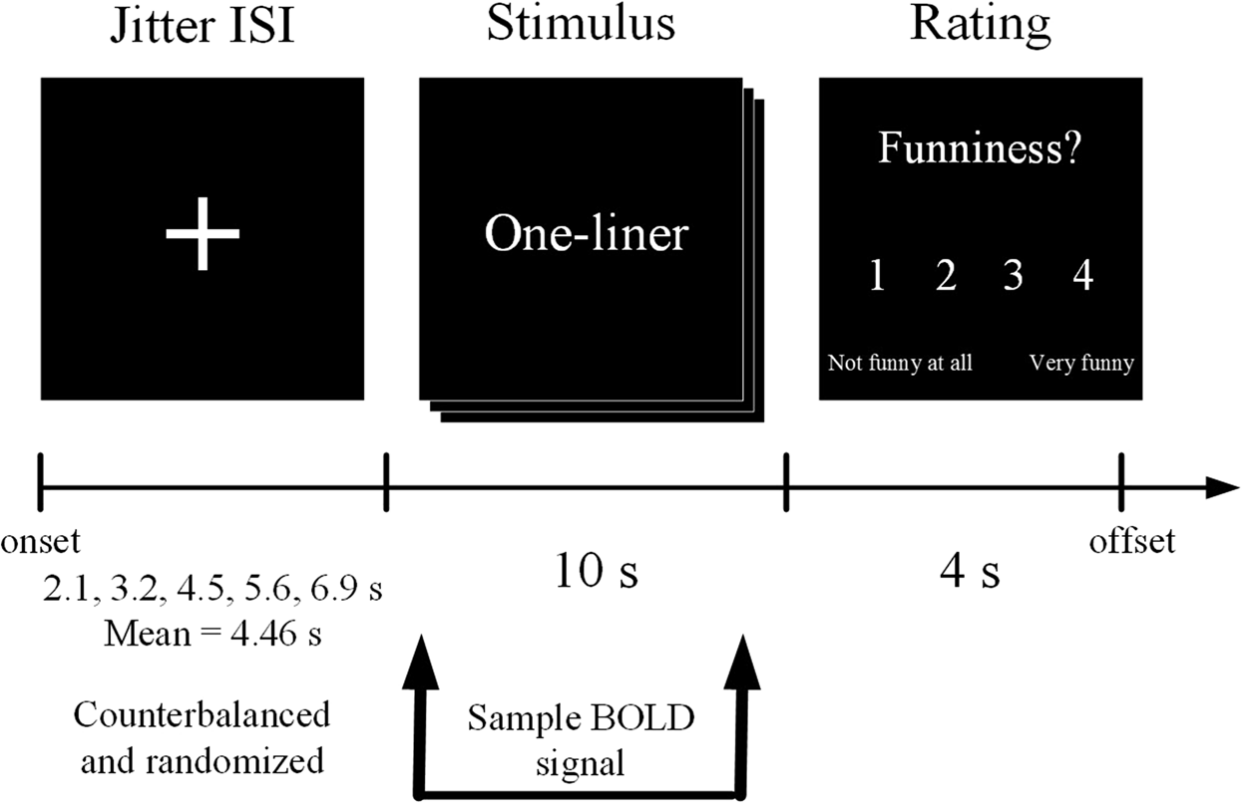
Experimental trial timeline. Each trial was shown for a mean jitter duration of 4.46 s. A one-liner stimulus was shown for a fixed duration of 10 s, followed by a 4-point rating scale, asking how funny the participant thought the humor was (1 = not funny at all to 4 = very funny).

The stimuli were presented in an event-related fMRI paradigm. We employed a 2 × 2 factorial design, with the factors humor motivation (benign and detrimental humor) and target (self and others). Stimuli were presented to participants using the PC-based stimulation package E-Prime 2.0 Software (Psychology Software Tools, Inc., Pittsburgh, PA), and all stimuli were presented in black and white. The study examined the neural correlates of the main effects and interactions between motivation and target. In each trial, the participant was first shown the fixation target for a jittered interstimulus interval (ISI), which was randomly varied among 2.1, 3.2, 4.5, 5.6, and 6.9 s and counterbalanced across stimulus styles. Each one-line sentence was shown once for 10 s. Participants provided a subjective funniness judgment by pressing one of four buttons on a keypad positioned under their right hand to indicate how funny they thought the stimuli was (1 = “not funny at all” to 4 = “very funny”), which lasted for 4 s (Fig. 6). Each participant read 100 one-line sentences (trials). Each of the five conditions (SE, AF, SD, AG and CON) consisted of 20 trials. There were a total of four functional runs with 25 trials per run. There were five stimuli from each of the five categories in each run and the stimuli were randomly placed within the run. The four runs in each sequence were presented in a counterbalanced order across participants. Each functional run lasted 7 min and 47.50 s, with a 2-min break between runs. The total duration of the experiment was approximately 37 min and 10 s per participant.

### Image acquisition

Functional and structural MRI was performed on a 3T scanner (Megnetom Skyra, Erlangen, Germany) using a standard 32-channel head coil at the Research Center for Mind, Brain & Learning in Taiwan. Participants viewed the projection screen via a mirror system placed on top of the head coil. Functional images were obtained using a single-shot gradient-echo, echo-planar imaging (EPI) sequence with the following parameters: TR = 2000 ms, TE = 30 ms, flip angle = 90°, 64 × 64 matrix, field of view (FOV) = 240 × 240 mm^2^, and voxel size = 3.75 × 3.75 × 3.70 mm^3^. Each EPI volume contained 36 transversal slices (3.7-mm-thick, no gap) in an interleaved order. Each run contained 232 functional images. To aid in localization of activation, we acquired high-resolution T1-weighted magnetization-prepared rapid gradient-echo imaging (MP-RAGE) 3-D MRI using the following pulse sequence: TR = 1900 ms, TE = 3.30 ms, flip angle = 9°, 256 × 256 matrix, FOV = 256 × 256 mm^2^, voxel size = 1 × 1 × 1 mm^3^ resolution, and 192 1-mm thick contiguous axial images.

### Image analysis

Functional images were preprocessed and statistically analyzed using Statistical Parametric Mapping software (SPM12; Wellcome Department of Cognitive Neurology, London, UK). The first three repetitions of each EPI series were discarded before image analyses to allow for T1 saturation effects. For preprocessing, the EPI data were corrected for slice time and head movement to the middle functional volume, co-registered and normalized to the standard Montreal Neurological Institute (MNI, McGill University, Montreal, Quebec, Canada) coordinate space and spatially smoothed using a Gaussian kernel with a full width at half maximum (FWHM) of 8 mm.

Statistical analyses were performed on single-subject and group data using a two-level general linear model (GLM) approach. For each subject and each condition, a comparison of interest was implemented as an individual contrast image. Each participant’s blood oxygen level-dependent (BOLD) signal was modeled with fixed-effects analysis that modeled the different conditions (SE, AF, SD, AG) as events using a canonical hemodynamic response function (HRF) with a temporal derivative. All six motion parameters were included as nuisance regressors in the generalized linear model (GLM). Each participant was analyzed for his or her responses to the humor styles compared to those to the control baseline stimuli (CON) for each condition using a GLM. These contrast images were used in a second-level analysis. Each participant’s contrast volumes were fed into a random-effects analysis, which created group average maps for all contrasts across the entire brain using a within-subject flexible factorial design. The modulation was analyzed using two-way analysis of variance (ANOVA), with the factors humor motivation (benign and detrimental humor) and target (self and others). The present study was focused on the main effects, interactions, and simple main effects.

ROI statistical analysis was performed for specific a priori hypotheses^36^. Based on previous studies of humor appreciation and laughter response studies^1,2,16–20^, the resulting mask of humor processing was associated with brain regions in the predefined ROI. The present study focused on nine ROIs in the NAc, midbrain, amygdala, TP, ACC, MTG, and PFC. Additionally, based on previous humor studies involving theory of mind^20^, our study included a TPJ region in the ROI for each contrast. ROI masks were constructed from the Wake Forest University (WFU) PickAtlas (www.fmri.wfubmc.edu/) and Marsbar (marsbar.sourceforge.net/). The boundaries of the NAc ROI were defined according to previous studies^37,38^. In this study, activations were considered significant at *p* < 0.05 and corrected for multiple comparisons for the familywise error rate (FWE) across the ROIs at the voxel level with a cluster size greater than or equal to 8 contiguous voxels after small volume correction (SVC) of anatomical ROIs. To visualize the signal change for significant brain regions, we extracted time courses from the beta values of peak voxels of the regions. We further conducted PPI analyses to investigate task-specific changes in functional connectivity between seed regions and other brain regions involved in humor appreciation. Based on previous humor studies^1,2,16–24,26–28^, PPI analyses primarily identified inter-regional interactions using the NAc, midbrain, TPJ, amygdala, and TP as the seed regions. Finally, the present study also conducted the analyses in BOLD activity correlation with self-defeating humor and subjective funniness ratings as well as affiliative humor and subjective funniness ratings.

## Electronic Supplementary Material

Supplementary Information
