# Supplementary material for: Appreciation of different styles of humor: An fMRI study
Source: Sci Rep. 2018 Oct 23;8:15649. doi: 10.1038/s41598-018-33715-1 (PMC6199304; doi:10.1038/s41598-018-33715-1)
Supplement: Supplementary file 1 — Supplementary Information [file 41598_2018_33715_MOESM1_ESM.pdf]

Appreciation of different styles of humor: An fMRI study

Yu-Chen Chan[1*,2], Wei-Chin Hsu[3], Yi-Jun Liao[4], Hsueh-Chih Chen[5, 6, 7], Cheng-Hao Tu[8], Ching-Lin Wu[6, 9]

[1]Department of Educational Psychology and Counseling, National Tsing Hua University, Hsinchu, Taiwan

[2]Research Center for Education and Mind Sciences, NTHU, Hsinchu, Taiwan

[3]Graduate Institute of Applied Science and Technology, National Taiwan University of Science and Technology, Taipei, Taiwan

[4]Institute of Learning Sciences and Technologies, National Tsing Hua University, Hsinchu, Taiwan

[5]Department of Educational Psychology and Counseling, National Taiwan Normal University, Taipei, Taiwan

[6]Institute for Research Excellence in Learning Sciences, NTNU, Taipei, Taiwan

[7]Chinese Language and Technology Center, NTNU, Taipei, Taiwan

[8]Graduate Institute of Acupuncture Science, China Medial University, Taichung, Taiwan

[9]Program of Learning Sciences, National Taiwan Normal University, Taipei, Taiwan

Supplementary Information

1. Four humor styles

The present study developed one-line humor stimuli based on the model of humor styles by Martin et al. The Humor Styles Questionnaire (HSQ) measures a multidimensional personality trait. However, the present study uses one-liner humor to replace the self-report HSQ, in order to investigate appreciation of humor designed to facilitate social relationships and enhance the self.

Four distinct humor styles emerge from the combination of two dimensions, motivation (benign and detrimental) and target (self and others). Therefore, sense of humor can be divided into the following styles: self-enhancing humor (SE; benign, self-directed humor used to enhance the self directly), affiliative humor (AF; benign, other-directed humor used to facilitate social relationships), self-defeating humor (SD; detrimental, self-directed humor used to facilitate social relationships at one's own expense), and aggressive humor (AG; detrimental, other-directed humor used to enhance the self at the expense of others) (Table S1).

Table S1 Four humor styles described by Martin et al.

Motivation	Target	
	Self	Others
Benign	***Self-enhancing humor (SE)***<br>• Enhancing the self<br>• Using benign humor for self	***Affiliative humor (AF)***<br>• Facilitating relationships<br>• Using benign humor for others
Detrimental	***Self-defeating humor (SD)***<br>• Facilitating relationships<br>• Using detrimental humor for self	***Aggressive humor (AG)***<br>• Enhancing the self<br>• Using detrimental humor for others

2. Stimuli

In contrast to the use of the self-report HSQ in previous studies, the present study developed 'humorous one-liners' and the matched stimuli, 'non-humorous one-liners' (baseline). The corresponding baseline non-humorous one-liner stimuli were constructed with neutral sentences of matching length and punctuation.

In our previous fMRI studies of verbal humor, the stimuli were verbal jokes composed of two components, the setup and punch line. The present study instead used "one-liner" humor using the exaggeration technique, which conceptually overlaps with nonsense or absurd humor. Using this technique, some element of a

situation was exaggerated in terms of quality or quantity to such an extreme as to violate common sense understanding.

Table 2 offers examples of the one-liner humor used in this study in the original Mandarin Chinese along with English translations. Some of the humor or funniness may have been lost in the translation to English. For example, in the third example, the idea of a dog barking at a ghost is drawn from Chinese culture, a situation that may make less sense for people from other cultures.

Table S2 Examples of one-liner humor and non-humor

Style	Example
SE	If each of my admirers were a strand of hair, I would need two heads.<br>若每個愛慕者都是我的一根頭髮，我需要兩顆頭。
AF	If each of your admirers were a strand of hair, you would need two heads.<br>若每個愛慕者都是你的一根頭髮，你需要兩顆頭。
SD	If each of my admirers were a strand of hair, I would be bald.<br>若每個愛慕者都是我的一根頭髮，我一定是禿頭。
AG	If each of your admirers were a strand of hair, you would be bald.<br>若每個愛慕者都是你的一根頭髮，你一定是禿頭。
CON	Time spent with family always flies, so we need to cherish it.<br>和家人相處的時間總是稍縱即逝，要好好地珍惜。
SE	There are only two things that I can do in my life – this and that.<br>我一生只會了兩件事：這個很會，那個很會。
AF	There are only two things that you can do in your life – this and that.<br>你一生只會了兩件事：這個很會，那個很會。
SD	There are only two things that I cannot do in my life – this and that.<br>我一生只不會兩件事：這也不會，那也不會。
AG	There are only two things that you cannot do in your life – this and that.<br>你一生只不會兩件事：這也不會，那也不會。
CON	There are two sports that I am best at – water ballet and golf.

	我最擅長的兩種運動：水上芭蕾、高爾夫球。
SE	The flowers in my painting are so fragrant; even bees can’t resist diving into them.<br>我畫的花像有香味般，連蜜蜂都忍不住撞上去。
AF	The flowers in your painting are so fragrant; even bees can’t resist diving into them.<br>你畫的花像有香味般，連蜜蜂都忍不住撞上去。
SD	My self-portrait looks like a ghost, even dogs want to bark when they see it.<br>我的自畫像像鬼附身，狗看到了都會開始狂吠。
AG	Your self-portrait looks like a ghost; even dogs want to bark when they see it.<br>你的自畫像像鬼附身，狗看到了都會開始狂吠。
CON	This self-portrait is vivid; the character’s charm is expressive.<br>這幅自畫像栩栩如生，人物的神韻是相當傳神。
SE	I have a great memory; I even remember the fingerprints of the hands I’ve shaken.<br>我記憶力超好，若跟我握過手，我連指紋都記得。
AF	You have a great memory; you even remember the fingerprints of the hands you’ve shaken.<br>你記憶力超好，若跟你握過手，你連指紋都記得。
SD	I have a terrible memory; I have to ask my father for his last name.<br>我記憶力真糟，有時我還會問我爸爸「您貴姓」。
AG	You have a terrible memory; you have to ask your father for his last name.<br>你記憶力真糟，有時你還會問你爸爸「您貴姓」。
CON	Mnemonics involves the use of imagery and memorable phrases to aid the memory.<br>用心像和口訣，輔助記憶的方法稱為「記憶術」。
SE	I am a born beauty; my skin hasn’t changed since I was 3 years old.<br>我天生麗質，三歲以後我臉上的皮膚就再也沒變過。
AF	You are a born beauty; your skin hasn’t changed since you were 3 years old.<br>你天生麗質，三歲以後你臉上的皮膚就再也沒變過。
SD	I am innocent and pure; my brain hasn’t changed since I was 3 years old.<br>我天真無邪，三歲以後我腦裡的東西就再也沒變過。
AG	You are innocent and pure; your brain hasn’t changed since you were 3 years old.<br>你天真無邪，三歲以後你腦裡的東西就再也沒變過。
CON	The scenery in these old towns has not changed in over a decade.<br>走訪舊城鎮，這裡的景色十多年來幾乎再也沒變過。

Note: SE = self-enhancing humor; AF = affiliative humor; SD = self-defeating humor;

AG = aggressive humor; CON = one-liner non-humor (control baseline)

3. Behavioral studies

We performed two behavioral studies to choose the stimuli. In the first behavioral study, 78 participants evaluated the stimuli using pencil and paper. Participants included 49 males and 29 females, ranging in age from 18 to 23 (19.71 ± 0.89). Participants rated one-liner humor and one-liner non-humorous stimuli. The material for each condition was 140 sentences, with a total of 700 sentences used. Participants rated stimuli printed on paper, classified the stimuli and rated the degree of comprehensibility, funniness, and exaggeration on a 9-point scale, with a higher score indicating a higher degree. We also controlled for gender content in the stimuli. If the content of one-liner humor was associated with gender differences, then two versions of the stimulus were used for males and females, and participants read the material corresponding to their genders.

The overall correct classification rate was 90.43%, indicating that participants could correctly judge the five types of stimuli. The comprehensibility was 8.32 ± 0.62, indicating that participants could perceive what the stimuli were intended to express. The overall average of funniness for one-liner humor was 5.26 ± 2.29, meaning that one-liner humor could indeed amuse our participants. According to the rating results, we chose 135 sentences to represent each type of stimuli, for a total of 675 sentences. The length of each sentence was 19 to 24 Chinese characters, the mean and standard

deviation of Chinese characters in a sentence was 21.77 ± 1.16 (Table S3).

Table S3 Category judgment and the mean and standard deviation for comprehensibility and funniness

	Category judgment (%)		Comprehensibility		Funniness	
Style	correct	error	*M*	*SD*	*M*	*SD*
SE	89.65%	10.35%	8.44	0.51	5.32	1.82
AF	84.98%	15.02%	8.25	0.68	4.65	1.86
SD	87.82%	12.18%	8.17	0.68	5.53	1.79
AG	92.35%	7.65%	8.41	0.56	5.55	1.76
CON	97.34%	2.66%	8.63	0.58	1.36	0.79

Note: using a 9-point scale. *M* = mean; *SD* = standard deviation; SE = self-enhancing humor; AF = affiliative humor; SD = self-defeating humor; AG = aggressive humor; CON = one-liner nonhumor

The second behavioral study had 63 participants, including 29 males and 34 females, ranging in age from 19 to 30 (22.06 ± 2.57). The experiment was programmed by E-Prime 2.0. Every participant rated 135 sentences, including sentences for four humor styles and one-liner nonhumor (baseline), for a total of five types of stimuli and 27 sentences for each style. The experimental design was a two

factors within-subject design. The independent variables were the motivation and the target of humor; both variables had two levels, benign or detrimental motivation, and humor toward self or others, SE, AF, SD, and AG. In addition, corresponding baseline one-liner nonhumor stimuli were constructed with neutral sentences (CON) of matching length and punctuation. Dependent variables included reaction time; stimulus categorizations; and rating the levels of comprehensibility, funniness, and exaggeration. Categorizing refers to classification of the humor. Comprehensibility denotes how well participants understand the stimuli. Funniness indicates how amused participants were. Exaggeration refers to how unexpected the one-liner stimuli were to participants. Participants rated the one-liner stimuli on a 7-point scale. Furthermore, since the display order of stimuli might have influenced the experimental results, we used a counterbalanced design to decrease the confounding effect of display order.

The procedure of the experiment was as follows. First, participants read the one-liner stimulus and pressed the space bar once they understand the stimulus. Participants then classified the stimuli and rated the levels of comprehensibility, funniness, and exaggeration on a 7-point scale. Participants inputted the rating on the number buttons of the keyboard, with larger numbers indicating higher levels. After rating was completed, the monitor displayed “break”. Once participants recovered,

they pressed the space bar to read the next stimulus and rating task. Participants took breaks after responding to each series of several sentences, prompted by the monitor displaying “take a rest”. Participants returned to the task by pressing the space bar.

An interval had 45 trials, and each stimulus style had 9 trials. The trials were assigned in a random interval. For every trial, the screen displayed a cross for 600 ms to notify participants that the trial was beginning. Then, a stimulus was displayed in the center of screen, and participants pressed the space bar at their self-paced reading time to rate the stimulus, including the degree of comprehensibility, funniness, and exaggeration. The screen displayed “break” after each rating task was completed. Participants continued to the next trial by pressing the space bar after emotional recovery. The screen displayed “The End” when all tasks were finished. The entire experiment took 60 minutes, including rest time.

The average response time for one-liner stimuli was 4932.84 ± 1931.22 (ms). The results of one-way repeated-measures ANOVA showed that the difference in comprehensibility was significant between one-liner humor and one-liner nonhumor, $F(4, 248) = 20.45$, $p < .001$; participants understood one-liner nonhumor better than one-liner humor. The difference in funniness was also significant, $F(4, 248) = 128.71$, $p < .001$; one-liner humor was funnier than one-liner nonhumor. The difference in exaggeration was also obvious, $F(4, 248) = 942.95$, $p < .001$; one-liner humor was

more exaggerated than one-liner nonhumor (Table S4).

Table S4 Reaction time of comprehensibility and the mean and standard deviation for comprehensibility, funniness and exaggeration.

	Reaction time (ms)		Comprehensibility		Funniness		Exaggeration	
Style	*M*	*SD*	*M*	*SD*	*M*	*SD*	*M*	*SD*
SE	4649.85	1778.77	6.52	0.50	4.29	1.13	5.83	0.62
AF	5384.56	2000.76	6.37	0.54	3.94	1.10	5.43	0.59
SD	5396.43	2025.98	6.34	0.59	4.45	1.05	5.55	0.69
AG	5160.98	1884.62	6.42	0.49	4.12	1.00	5.55	0.67
CON	4072.38	1659.21	6.67	0.39	1.96	0.99	1.70	0.77

Note: using a 7-point scale. *M* = mean; *SD* = standard deviation; SE = self-enhancing humor; AF = affiliative humor; SD = self-defeating humor; AG = aggressive humor; CON = one-liner nonhumor

Table S5 lists the results of classification, and a Chi-square test showed that the percentage of the categories checked was significantly different among the five types of stimuli, $\chi^2(20) = 28723.702$, $p < .001$. A post hoc test shows that participants correctly classified all five types of stimuli.

Table S5 Percentage of category judgment

Choice of category	Style				
	SE	AF	SD	AG	CON
SE	92.89%	0.88%	4.12%	0.18%	0.88%
AF	1.18%	90.24%	0.41%	2.76%	1.76%
SD	4.12%	0.35%	91.18%	1.00%	0.35%
AG	0.24%	5.29%	2.29%	94.06%	1.06%
CON	0.53%	1.18%	0.65%	0.76%	95.06%
Loss of judgment	1.06%	2.06%	1.35%	1.23%	0.88%

Note: SE = self-enhancing humor; AF = affiliative humor; SD = self-defeating humor; AG = aggressive humor; CON = one-liner nonhumor

4. **Post-scan rating results**

4.1 Results of comprehensibility and funniness ratings

The mean and standard deviation for comprehensibility were 6.71 ± 0.50, indicating that all stimuli (humor and nonhumor) were comprehensible to participants. The mean funniness rating for both humor types was 4.37 ± 1.06. One-way repeated-measures ANOVA performed on participants' funniness ratings was significant, *F*(4,

164) = 99.72, $p < .001$, $\eta_p^2 = .71$, and Bonferroni post hoc tests revealed that the humor conditions were significantly funnier than the nonhumor condition.

Table S6 Mean and standard deviation for comprehensibility and funniness during post-scan ratings

Styles	Comprehensibility		Funniness	
	M	*SD*	*M*	*SD*
Self-enhancing humor (SE)	6.74	0.49	4.43	1.12
Affiliative humor (AF)	6.63	0.50	4.08	1.03
Self-defeating humor (SD)	6.64	0.54	4.74	0.94
Aggressive humor (AG)	6.68	0.49	4.24	1.06
Nonhumor (CON)	6.85	0.44	1.83	1.12

Note: *M* = mean; *SD* = standard deviation

4.2 Post-scan ratings: Category classification

Pearson's Chi-square analysis revealed a significant relationship between stimulus type and category judgment, $\chi^2(16) = 13527$, $p < .001$, indicating a significant difference in category judgment across the five conditions. For example, In terms of self-enhancing humor, the percentage of 'SE' category judgments was

93.5%, ‘AF’ was 1.3%, ‘SD’ was 3.7%, ‘AG’ was 0.001%, and ‘CON’ was 1.1% for SE humor. Post hoc tests revealed that significantly more self-enhancing humor stimuli were present in the self-enhancing humor condition (‘SE’ = 93.5%) than in the other four conditions. The percentage of affiliative humor was 88.2%, self-defeating humor was 88.9%, aggressive humor was 90.0%, and the neutral control was 97.7%.

Table S7 Percentages of category judgments during post-scan ratings

	Benign humor				Detrimental humor						Total
	SE	%	AF	%	SD	%	AG	%	CON	%	
SE	**785**	93.5	11	2.1	31	7.7	4	-	9	-	840
AF	18	1.3	**741**	88.2	9	-	50	6.4	22	0.8	840
SD	65	3.7	4	1.1	**747**	88.9	18	2.3	6	-	840
AG	1	-	54	6.0	19	2.1	**756**	90.0	10	0.8	840
CON	3	1.1	7	2.5	2	0.7	7	1.2	**821**	97.7	840
Total	872		817		808		835		868		4200

Note: SE = self-enhancing humor; AF = affiliative humor; SD = self-defeating humor; AG = aggressive humor; CON = one-liner nonhumor
